# Structural drivers and social protection: mechanisms of HIV risk and HIV prevention for South African adolescents

**DOI:** 10.7448/IAS.19.1.20646

**Published:** 2016-04-15

**Authors:** Lucie Dale Cluver, Frederick Mark Orkin, Franziska Meinck, Mark Edward Boyes, Lorraine Sherr

**Affiliations:** 1 Department of Social Policy and Intervention, Oxford University, Oxford, UK; 2 Department of Psychiatry and Mental Health, University of Cape Town, Cape Town, South Africa; 3 DPHRU, School of Clinical Medicine and DST-NRF Centre of Excellence in Human Development, University of the Witwatersrand, Johannesburg, South Africa; 4 School of Psychology and Speech Pathology, Curtin University, Perth, Australia; 5 Research Department of Infection and Population Health, University College Medical School, UCL, London, UK

**Keywords:** prevention, adolescents, social protection, abuse, education, poverty

## Abstract

**Introduction:**

Social protection is high on the HIV-prevention agenda for youth in sub-Saharan Africa. However, questions remain: How do unconditional cash transfers work? What is the effect of augmenting cash provision with social care? And can “cash plus care” social protection reduce risks for adolescents most vulnerable to infection? This study tackles these questions by first identifying mediated pathways to adolescent HIV risks and then examining potential main and moderating effects of social protection in South Africa.

**Methods:**

This study was a prospective observational study of 3515 10-to-17-year-olds (56.7% female; 96.8% one-year retention). Within randomly selected census areas in four rural and urban districts in two South African provinces, all homes with a resident adolescent were sampled between 2009/2010 and 2011/2012. Measures included 1) potential structural drivers of HIV infection such as poverty and community violence; 2) HIV risk behaviours; 3) hypothesized psychosocial mediating factors; and 4) types of social protection involving cash and care. Using gender-disaggregated analyses, longitudinal mediation models were tested for potential main and moderating effects of social protection.

**Results:**

Structural drivers were associated with increased onset of adolescent HIV risk behaviour (*p*<0.001, *B*=0.06, SE=0.01), fully mediated by increased psychosocial problems. Both cash and care aspects of social protection were associated with reductions in HIV risk behaviour and psychosocial deprivations. In addition, cash social protection moderated risk pathways: for adolescent girls and boys experiencing more acute structural deprivation, social protection had the greatest associations with HIV risk prevention (e.g. moderation effects for girls: *B*=−0.08, *p*<0.002 between structural deprivation and psychosocial problems, and *B*=−0.07, *p*<0.001 between psychosocial problems and HIV risk behaviour).

**Conclusions:**

Adolescents with the greatest structural deprivation are at higher risk of HIV, but social protection has the greatest prevention effects for the most vulnerable. Social protection comprising unconditional cash plus care was associated with reduced risk pathways through moderation and main effects, respectively. Our findings suggest the importance of social protection within a combination package of HIV-prevention approaches.

## Introduction

Social protection is a new focus of HIV-prevention efforts in sub-Saharan Africa, supported by international agencies such as UNICEF, UNAIDS, the World Bank and PEPFAR-USAID and increasing political commitment within the region [1–4]. There is strong evidence that HIV-infection rates are increased by structural drivers [5] including violence [6], parental HIV/AIDS [7], food insecurity [8] and informal housing [9]. Aiming to address these drivers, unconditional government cash transfers and transfers conditional on education have been shown to reduce HIV risk behaviour and HIV infection in studies in South Africa, Kenya and Malawi [10–14].

New research also shows that combining psychosocial and economic social protection provision (“cash plus care”) may further reduce vertical and horizontal HIV risks [15–17]. This reflects evidence from studies of social and emotional development in adolescence, where support from families or other caring adults has been found essential for a range of healthy behaviours [18]. In particular, provision of warmth and supervision/monitoring from primary caregivers may be linked to adolescent sexual risk reduction [19].

However, there remain important unanswered questions about social protection as an HIV-prevention tool among youth. First, how do unconditional cash transfers and other types of social protection such as psychosocial care reduce HIV risks? In order to refine prevention policies, it is essential to understand the mechanisms by which they may impact high-risk populations [20].

A second unanswered question, essential for prevention programming, is whether social protection is effective for the most vulnerable adolescents. Policies recognize the importance of reaching key populations with prevention technologies. However, the same psychological, economic and social barriers that contribute to adolescents’ hyper-vulnerability to HIV can also reduce their capacity to access and utilize existing prevention interventions. It is important to test whether social protection can interrupt the impacts of multiple structural drivers on key populations.

This study examines the impacts of social protection delivered by governments, NGOs and family, in real-world low-resource conditions in South Africa. It tests 1) the potential pathways from structural disadvantage to adolescent HIV risks and 2) the nature and 3) the extent of the effects of cash and care types of social protection on adolescent HIV risk pathways.

## Methods

### Participants and procedures

For this study, 3516 adolescents aged 10 to 17 (56.7% female, no exclusion criteria) were interviewed in 2009/2010 and followed up a year later (2011/2012). The retention rate was 96.8% and the baseline refusal rate <2.5%. Within two South African provinces, Mpumalanga and the Western Cape, two urban and two rural health districts with >30% antenatal HIV prevalence were selected. Within each health district, census enumeration or tribal authority areas were randomly sampled until sample size was attained. Every household was visited in each area and was included in the study if they had a resident adolescent. One randomly selected adolescent per household was interviewed individually for 60 to 70 minutes in the language of their choice. Questionnaires and consent forms were translated and checked with back-translation into Xhosa, Zulu, Sotho, Swati and Tsonga.

Ethical protocols were approved by three university IRBs: Oxford, Cape Town and KwaZulu-Natal, and by provincial health and education departments. Voluntary written informed consent was obtained from adolescents and primary caregivers, with all consent procedures read aloud in cases of limited literacy. No incentives were given, apart from refreshments and certificates of participation. The interviewers were trained in working with vulnerable youth, and confidentiality was maintained, except where there was risk of significant harm or where assistance was requested. Where participants reported recent abuse, rape or risk of significant harm, referrals were made to child protection, HIV/AIDS and health services, with follow-up support.

### Measures

All measures were completed by adolescents and were piloted with a group of 20 South African adolescents prior to use.

*Adolescent HIV risk behaviours* were measured at baseline and follow-up, using scales from the SA Demographic and Health Survey and the National Survey of HIV and Sexual Behaviour amongst Young South Africans [21,22]. Risk behaviours were all based on systematic review or strong epidemiological evidence of strong associations to increased HIV infection among youth in Southern Africa. *Transactional sex* was sex in exchange for school fees, food, shelter, transport or money; *age-disparate sex* comprised a sexual partner more than five years older than the adolescent [21]; *past-year initiation of sexual activity* was first having vaginal/anal sex in the past 12 months (with a partner of either gender); *unprotected sex* was inconsistent or no condom use (with any partner) when having sex in the past year; *multiple sexual partners* was having two or more past-year partners [9]; *casual partners* was having sexual partners who were not regular boyfriends/girlfriends; *sex whilst using substances* was having sex whilst drunk or using drugs. *Pregnancy* was becoming pregnant (girls) or making someone pregnant (boys). Baseline HIV risk behaviours were controlled for in all analyses.

*Structural drivers* were measured at baseline for inclusion in the predictive model. *Food insecurity* was measured using items from the National Food Consumption Survey [22] and determined as insufficient food for more than two days in the past week. *Formal/informal housing* used an item adapted from the South African census. *AIDS orphanhood* or *living with AIDS-unwell caregivers* (“AIDS-affected”): Given the low levels of HIV status knowledge and testing, parental AIDS illness and death were determined using verbal autopsy methods [23], validated in previous South African studies (sensitivity 89%; specificity 93% [24]). In this study, determination of HIV/AIDS required reported HIV+ status or a conservative threshold of three or more AIDS-defining illnesses, for example, Kaposi's sarcoma or shingles. *Community violence exposure* used the Child Exposure to Community Violence Checklist [20], adapted to the four most common community traumas for children in South Africa [21], coded as victimization by any of the following: assault, robbery and witnessing of stabbings and/or shootings.

*Potential psychosocial mediators* were measured at baseline and follow-up. *School non-enrolment* was school dropout due to any cause. *Alcohol and drug (substance) misuse* were measured using 15 items adapted from the National Survey of HIV and Risk Behaviour [25] and included regular (weekly or more often) alcohol use, inebriation and any drug use such as marijuana, Mandrax or crystal methamphetamine. *Adolescent behaviour problems* used the Child Behaviour Checklist [26] with established reliability and validity in multiple countries [26,27]. *Child abuse* included physical, emotional and sexual abuse and was measured using UNICEF scales designed for sub-Saharan Africa [28]. *Mental health distress* included depression (using the Children's Depression Inventory Short Form [29]), anxiety (using the Revised Children's Manifest Anxiety Scale [25], validated in South Africa [30]) and suicidality (using the Mini International Neuropsychiatric Interview for Children and Adolescents [31]). For each potential mediator item, the mean between the baseline and follow-up value was used [32].

*Social protection*. Types of social protection were identified in consultation with the South African National Departments of Social Development, Basic Education and Health; UNICEF; PEPFAR-USAID; Save the Children and with our teen advisory group of adolescents. Cash or cash-in-kind was measured at baseline and follow-up. *Child-focused cash transfer* was household access to either a government child support or foster child grant [33]. *Free schooling* was measured as free school and textbooks (as some “free” schools charge fees for books and other school necessities). *School feeding* indicated free, daily meals provided at school; *access to food gardens* was receiving food from a school or community garden. *Free school transport* and *free school uniform* were measured. *Food parcels* and *soup kitchen feeding* measured at least monthly provision. Care was also measured at baseline and follow-up. The Alabama Parenting Questionnaire [34] was used to measure *positive parenting* (e.g. primary caregiver praise and warmth) and *good parental monitoring* (e.g. household rules and consistent monitoring), used in South African studies of parenting programmes [35,36]. *Teacher social support* used a standardized scale for social, practical and emotional support from a teacher [37]. *Home-based carer support* (at least monthly household visits providing medical and social support) and *school counsellor access* were measured. Evidence suggests that in order to show effects, social protection requires sustained and predictable duration [38]. Consequently, each type of cash/cash-in-kind and care was coded positively if received at both baseline and one-year follow-up.

*Potential confounders of HIV risk and social protection access* were included as covariates in all models. 1) Confounders of HIV risk: *Baseline HIV risk behaviour* used the first-factor score from a factor analysis of the adolescent HIV risk behaviours above (35% variance explained); *adolescent age* and *gender* were asked. *HIV prevention knowledge* at baseline and follow-up was measured using a “free-listing” approach, asking “Can you write any things you think a person can do to avoid getting HIV or AIDS?” (as provision of pre-existing lists can overestimate knowledge levels). Scores were calculated by summed accurate methods (e.g. “use a condom”), minus summed inaccurate methods (e.g. “do not share food with an HIV-positive person”) with a range of −6 to +6. 2) Confounders of social protection access: *Urban/rural location* and *province* were identified. *Child migration* was measured as movement between households. *Number of children in the household* was measured using a household “map,” which identified ages and genders of all those sleeping in the dwelling. *Female primary caregiver* was identified as “the person who lives with you and looks after you most.” *Adolescent access to birth certificate* was included as required documentation for some services.

### Analyses

Analyses were disaggregated by gender and conducted in six stages on the longitudinal sample of adolescents with data from both time points (*n*=3401). First, each grouping of potential structural drivers, potential cash/cash-in-kind and care moderators, and follow-up adolescent risk behaviours was factor-analyzed in SPSS v21. This study aimed to examine the moderation of possibly mediated relationships: to examine patterns among the five constructs of potential structural drivers, psychosocial mediators, social protection (cash/cash-in-kind and care) moderators and HIV outcomes, the respective variables were combined – with their relative weights according to factor analysis – into the corresponding constructs. First-factor scores explained 32% of variance for structural drivers, 35% for cash/cash-in-kind, 39% for care and 31% for HIV risk and in each instance were considered suitable for use as factor-scored constructs across all the respective contributing variables. Potential psychosocial mediators were standardized. Second, because few adolescents below 12 years old reported any sexual activity (*n*=9), the dataset was limited to adolescents aged 12 to 18 at follow-up, excluding 305 boys and 428 girls from further analyses and yielding *n*=2668. Additionally, types of social protection that reached <100 adolescents were excluded from analyses due to low cell sizes and disaggregation by gender of all models. These were food parcels (0.1%, *n*=3), soup kitchen (0.4%, *n*=10), free school transport (0.9%, *n*=8), free school uniform (0.6%, *n*=7), home-based caregiver (0.7%, *n*=18) and school counsellor (3.7%, *n*=98). Seven types of cash/cash-in-kind and care social protection remained: child grant (55.7% of adolescents, *n=*1486), free school and textbooks (72.6%, *n*=2552), free school meals (72.3%, *n*=1930) and food gardens (4.9%, *n*=132), positive parenting (24.9%, *n*=664), good parental monitoring (22.2%, *n*=779) and teacher social support (7.9%, *n*=211).

**Figure 1a and b:**
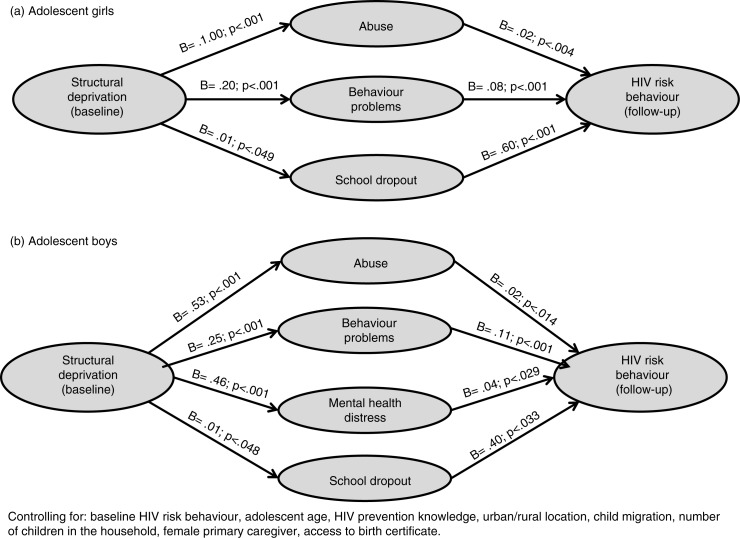
Mediating effects of psychosocial factors on associations between structural deprivation and subsequent HIV risk behaviour among adolescents.

Third, in order to determine whether it was valid to test more complex pathways, initial regression models checked whether there were associations between structural drivers and adolescent HIV risk behaviours one year later, controlling for baseline HIV risk behaviour, age, gender and HIV knowledge. Fourth, hypothesized risk pathways were tested for each gender (Figure 1), using Hayes’ PROCESS macro in SPSS that allows simultaneous testing of multiple and moderated mediation [39]. Potential direct and indirect pathways from baseline structural deprivation via each potential psychosocial mediator to incident HIV risk behaviour were tested using Hayes’ Model 4, controlling for the covariates mentioned above.

**Figure 2a and b:**
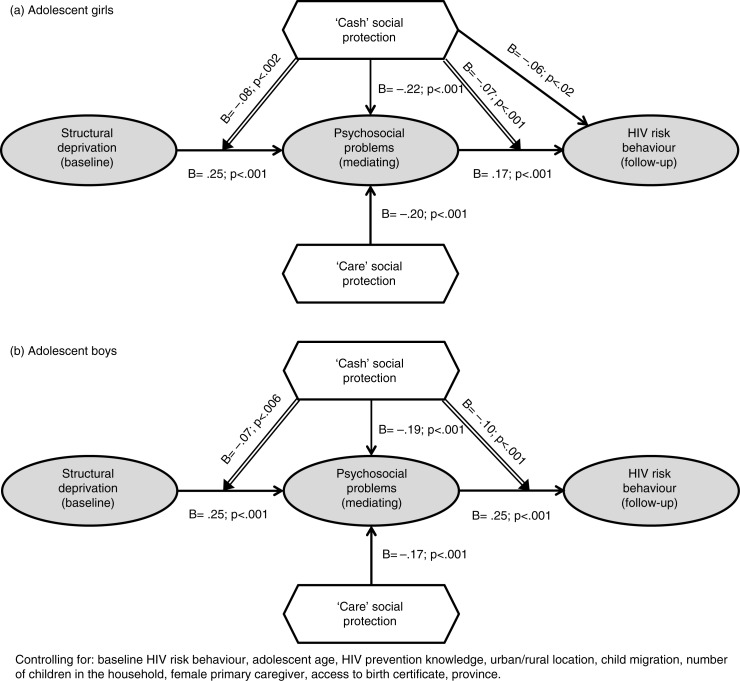
Main and moderating effects of cash/cash-in-kind and care social protection on mediated pathways to HIV risk behaviour among adolescents.

Fifth, only those psychosocial factors that showed significant mediation were factor-analyzed together and the first factor score (variance explained: 43% girls and 41% boys), was used as a composite mediator scale for each gender. This simplified mediation model was then tested for adolescent boys and girls, in order to provide a base for testing the potential effects of social protection. Sixth, in order to test the potential effects of social protection, gender-disaggregated PROCESS models simultaneously tested the main and moderating effects of cash/cash-in-kind and care on the significant HIV risk pathways, controlling for the confounders mentioned. This process used Hayes’ Model 76 to allow testing of potentially dual moderation of each linkage within the mediation model [40]. For the final values of coefficients that are reported (Figure 2), the moderated mediation models were re-run using only significant covariates, namely baseline HIV risk behaviours, province and adolescent age.

Sixth, the effect on HIV risk behaviour via the psychosocial mediators of indicative changes (±1 s.d.) in the antecedent, structural deprivation, and the moderator, cash/cash-in-kind, was calculated and displayed, following Hayes [39], by substituting the significant coefficients yielded by PROCESS into successive regression equations:

1 $$m=i_{1}+a_{1}x+a_{2}w+a_{3}xw+a_{4}V+a_{i}D_{i}+e_{m}$$

For each case, *m* is the value of the mediator (psychosocial problems); *i*_1_ is the intercept; *a*_1_, *a*_2_ and *a*_3_ are the coefficients for the antecedent *x* (structural deprivation). The moderator is *w* (cash/cash-in-kind) and the first moderation term is *xw*; *a*_4_ is the coefficient for *V* (care); *a*_*i*_ *D*_*i*,_ for *i=*5 to 7, are the coefficients and mean values of the covariates age, province and baseline HIV risk behaviour; and *e*_*m*_ is the error in *m*. The value of the mediator *m* then carries into the second regression equation (2):

2 $$y=i_{2}+b_{1}m+b_{2}w+b_{3}mw+b_{4}V+b_{i}D_{i}+e_{y}+c_{3}x$$

For each case, *y* is the value of the outcome (HIV risk behaviour); *i*_2_ is the intercept; *b*_1_, *b*_2_ and *b*_3_ are the coefficients for mediator *m* (psychosocial problems), moderator *w* (cash/cash-in-kind) and the second moderation term *mw*; *b*_4_ is the coefficient for *V* (care). The expression *b*_*i*_ *D*_*i*_, for *i=*5 to 7, represents the coefficients and mean values of the covariates age, province and baseline HIV risk behaviour; and *e*_*y*_ is the error in *y*. The last term in (2), *c*_3_ *x*, represents the direct effect from antecedent deprivation to outcome HIV risk in the applicable Hayes model, but its size is negligible and it is insignificant, indicating “full mediation” as a result of the moderation.

## Results

### Structural deprivations, psychosocial consequences and HIV risk behaviours

Table 1 shows adolescent exposure to structural, psychosocial and HIV risk behaviours, by gender. Exposure to structural deprivation was high overall: 25.1% lacked sufficient food, 30.7% lived in informal housing, 47.2% were exposed to community violence and 40.1% were AIDS-affected (i.e. orphaned by AIDS or living with an AIDS-ill primary caregiver). Potential psychosocial mediators included 5.9% behaviour problems, 53.8% child abuse, 30% substance abuse, 6.4% school dropout and 29.4% clinical-level mental health distress. HIV risk behaviours included 3.3% for transactional sex, 2.8% for age-disparate sex, 14.9% for debut in the past year, 12.3% for inconsistent/no condom use, 2.1% for casual sex, 11.4% for multiple partners, 3.3% for sex while using substances and 2.9% for pregnancy. Some social protection factors showed very low access rates (home-based carer, school counsellor, soup kitchens, food parcels, free school transport or uniform – all under 100 adolescent recipients). The remaining factors were care (7.9% teacher support, 24.9% positive parenting and 21.4% good parental monitoring/supervision) and cash/cash-in-kind (55.7% child cash transfer, 5.1% pension, 72.3% free school meals, 74.4% free school textbooks and 4.9% food garden access).

**Table 1:** Number and proportion of adolescents experiencing structural deprivation, psychosocial problems and HIV risk and those receiving types of social protection

	Whole sample (*n*=2668)	Boys (*n* = 1170)	Girls (*n*=1498)	*p*
Any structural deprivation				
Food insufficiency	25.1% (671)	22.7% (266)	27.0% (405)	0.012
Informal housing	30.7% (830)	31.4% (471)	29.8% (349)	0.375
Community violence exposure	47.2% (1258)	50.7% (593)	44.4% (665)	0.001
AIDS-affected	31.7% (847)	28.5% (333)	34.3% (514)	0.001
Any psychosocial consequences				
Behaviour problems	5.9% (158)	8.4% (98)	4.0% (60)	0.001
Child abuse victimization	53.8% (1435)	51.1% (598)	56.7% (850)	0.001
Substance use	30.0% (801)	31.8% (372)	28.6% (429)	0.046
School dropout	6.4% (171)	5.0% (59)	7.5% (112)	0.023
Mental health risks	29.4% (786)	23.2% (271)	34.4% (515)	0.001
Any HIV risk behaviour				
Transactional sex	3.3% (88)	2.6% (30)	3.9% (58)	0.064
Age-disparate sex	2.8% (76)	2.6% (31)	3.0% (45)	0.640
Sexual debut last year	14.8% (394)	16.4% (192)	13.5% (202)	0.037
Inconsistent/no condom use	12.3% (329)	13.1% (153)	11.7% (176)	0.313
Casual sex	2.1% (56)	4.4% (51)	0.3% (5)	0.001
Multiple partners	11.4% (305)	15.8% (185)	8.0% (120)	0.001
Substance use while having sex	3.3% (88)	4.4% (52)	2.4% (36)	0.004
Pregnancy	2.9% (77)	0.5% (6)	4.7% (71)	0.001
Any care provision				
Home-based carer support	0.7% (18)	0.3% (3)	1.0% (15)	0.020
Teacher social support	7.9% (211)	7.7% (90)	8.1% (121)	0.715
School counsellor	3.7% (98)	3.7% (43)	3.7% (55)	0.996
Positive parenting	24.9% (664)	24.6% (288)	25.1% 376	0.774
Good parental monitoring/supervision	21.4% (572)	18.3% (214)	23.9% (358)	0.001
Any cash/cash-in-kind provision				
Child grant	55.7% (1486)	54.2 (634)	56.9% (852)	0.169
Food parcel	0.1% (3)	0.1% (1)	0.1% (2)	1.000
Soup kitchen	0.4% (10)	0.2% (2)	0.5% (8)	0.201
Pension	5.1% (136)	4.7% (55)	5.4% (81)	0.426
Free school meal	72.3% (1930)	74.0% (866)	71.0% (1064)	0.089
Free text books	74.4% (1984)	74.4% (870)	74.4% (1114)	1.000
Free school transport	0.8% (22)	0.8% (9)	0.9% (13)	0.832
Free school uniform	0.7% (19)	0.6% (7)	0.8% (12)	0.646
Food garden	4.99% (132)	5.6% (65)	4.5% (67)	0.209
Confounders				
Age	14.24 (SD 1.65) SE 0.03	14.17 (SD 1.63) SE 0.05	14.29 (SD 1.66) SE 0.04	0.056
Rural location	50.9% (1359)	49.4% (578)	52.1% (781)	0.172
Province MP	49.0% (1308)	50.6% (592)	47.8% (716)	0.160
HIV knowledge	1.14 (SD 0.63) SE 0.01	1.08 (SD 0.61) SE 0.02	1.14 (SD 0.65) SE 0.17	0.019
Baseline HIV risk behaviour	15.7% (418)	15.2% (178)	16% (240)	0.952

### Pathways from structural disadvantage to HIV risks

A preliminary check in regression confirmed that the structural drivers (factor-scored) significantly predicted HIV risk behaviour a year later (*B*=0.06, SE=0.01; *p*<0.001), controlling for potential confounders of baseline HIV risk, age, gender, HIV knowledge, urban/rural location, province, migration, numbers of children in the household, gender of primary caregiver and birth certificate access. Subsequently, separately for girls and boys, a PROCESS model tested simultaneous potential psychosocial mediators. Figure 1 shows B and *p*-values for direct and indirect pathways from structural drivers to the onset of adolescent HIV risk behaviours via each potential psychosocial mediator (controlling for all potential confounders) for girls and boys. For girls, the relationship between structural drivers and HIV risk behaviours was fully mediated by abuse, behaviour problems and school dropout, meaning that pathways from structural drivers to adolescent HIV risk were acting via increasing these psychosocial deprivations, which then in turn impacted HIV risk behaviours. Alcohol/drug use and mental health were not significant mediators. For boys, the relationship between structural drivers and HIV risk behaviours was fully mediated by abuse, mental health distress, behaviour problems and school dropout. Alcohol or drug use was not a significant mediator. For each gender, a composite scale of the significant psychosocial mediators comprised the first factor score of a factor analysis.

### Social protection mechanisms

Next, the effects of cash/cash-in-kind and care social protection provisions were tested as potential moderators of the mediated pathways from structural drivers to the onset of adolescent HIV risk behaviours. As two additional checks for robustness of findings, the models for girls and boys were run with only covariates that were significant (baseline HIV risk, age, province), and differences were negligible. Figure 2a shows B and *p*-values for direct, mediated and moderated pathways from structural drivers to adolescent HIV risks for girls and boys. Among girls, cash/cash-in-kind and care directly reduced psychosocial problems and cash directly reduced HIV risk behaviours (shown by arrows leading directly into the psychosocial and HIV risk constructs). In addition, for girls cash/cash-in-kind moderated the pathway from structural drivers to psychosocial problems and the pathway from psychosocial problems to HIV risk behaviour (shown by arrows leading into the pathways between these constructs, with stronger effects for those at highest structural and psychosocial risk). Thus, cash/cash-in-kind and care had the main effect of reducing HIV risk for all girls, but additionally cash/cash-in-kind showed greater effects for girls experiencing the most severe structural deprivation and psychosocial problems.

Among boys, cash/cash-in-kind and care both had the main effect of reducing psychosocial problems. Additionally, cash/cash-in-kind moderated the pathway from structural drivers to psychosocial problems and also moderated the pathway from psychosocial problems to HIV risk behaviour. Thus, cash/cash-in-kind and care had direct effects on reducing HIV risk for all boys, and cash/cash-in-kind additionally showed a greater effect for boys experiencing the most severe structural deprivation and psychosocial problems.

**Figure 3:**
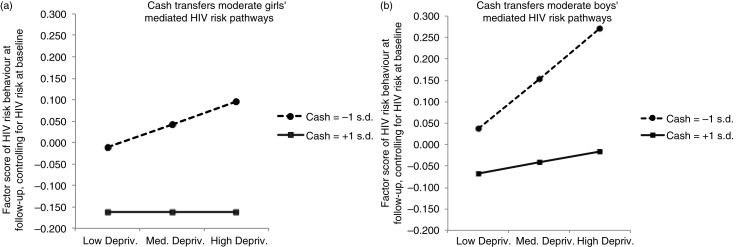
Moderated mediation for (a) adolescent girls and (b) adolescent boys: Structural deprivation to HIV risk behaviour via the mediator of psychosocial problems, with both pathways moderated by cash/cash-in-kind social protection.

Figure 3a for girls and Figure 3b for boys demonstrate the impact of cash/cash-in-kind on moderating the mediated HIV risk pathway for adolescent girls and boys, obtained by applying PROCESS outputs in equations (1) and (2) above. The dashed lines show adolescents in families receiving lower levels of cash/cash-in-kind social protection (+/−1 s.d. from the mean factor score). At all stages their HIV risk behaviour is higher, but it notably rises (for both adolescent girls and boys) as structural deprivation and the consequent psychosocial problems increase. In contrast, the continuous lines show adolescents in families receiving higher levels of cash/cash-in-kind social protection (+1 s.d. from the mean factor score). Their HIV risk behaviour remains lower and shows no or negligible rise as structural and psychosocial deprivation increases.

## Discussion and conclusions

This study has a number of important implications. Our findings show that adolescent HIV risks in South Africa are strongly driven by structural drivers (living in poverty, with AIDS-affected parents and in high-violence or informal communities) and that this association is mediated by (or occurs via) increased psychosocial problems (higher rates of abuse, mental health distress and school dropout). Through these pathways, incidence of adolescent HIV risk behaviour increases for both girls and boys.

In contrast, unconditional government cash transfers and psychosocial care showed associations with risk reduction, by directly mitigating the onset of adolescent HIV risk behaviour and the psychosocial mediators of HIV risk. Cash/cash-in-kind and care showed simultaneous associations in reducing different HIV risk pathways, independent of each other and of confounding factors. These findings support recent evidence from Uganda [41,42], suggesting that a combination social protection response may be more effective than single-intervention programming. It was notable in the present study that, for some care protections in particular, reach was very low and the existing provision could be valuably scaled up.

Finally, our findings show that social protection is most effective for adolescents for whom structural and psychosocial drivers place them at highest HIV risk – by moderating the damaging effects of structural deprivation on psychosocial risk and by moderating the damaging effects of psychosocial risk on HIV risk. Thus, cash plus care can potentially reach those who are most in need of prevention.

All social protection interventions tested in this study were existing government, NGO or family provisions, and all were unconditional in their provision. Thus, the findings of this study may have implications for the current debate between unconditional social protection approaches and cash incentive programmes based on direct sexual outcomes such as pregnancy or STI infection [43]. This study shows that adolescent HIV risk behaviours in South Africa are not driven solely by behavioural choice; instead their incidence is associated with structural and consequent psychosocial deprivation, which can be alleviated by unconditional cash/cash-in-kind and psychosocial care. Thus, incentive-based cash interventions that are premised on an understanding that adolescent sexual risk is primarily a conscious behavioural choice may fail those who are at the greatest structural risk of HIV infection.

Indeed, one of the mediating factors, child abuse, suggests an important consideration for cash incentive programmes that are conditional on adolescents remaining STI or HIV-free [43,44]. In this sample, 9.4% of adolescent girls and 5.1% of boys reported sexual abuse or rape, and child abuse was strongly associated with structural deprivation. Thus withdrawing cash or cash-in-kind provision from adolescents who become infected may re-victimize them and potentially increase rather than decrease structural risk pathways.

Other conditional cash programmes are premised on educational attendance [45,46]. These findings suggest that this pathway is a strong predictor of HIV risk behaviour onset and that unconditional cash provision was associated with reductions in similar risk pathways to education-conditioned programmes. Further research is clearly required to test whether education-conditional or -unconditional programmes have a greater impact on the education pathways to HIV risk. To date, evidence is mixed: in Malawi there were no differences between the two on HIV risk [10], and in Zimbabwe there were no differences among pre-adolescents, but education conditionality increased school attendance more among adolescents [47]. Two new studies of conditional cash transfers in South Africa showed no differences in HIV-infection rates between transfers for young women conditioned on education attendance and those conditioned on a range of extracurricular and health-seeking behaviours. However, the latter did show reductions in HSV-2 infections, and both showed consistent protective impacts of educational attendance on HIV-infection risk [46,48]. Overall, it is an essential question whether the added costs and administrative requirements of conditionality (e.g. monitoring, enforcing sanctions) are balanced by additional risk reduction benefits.

This study has a number of limitations. Non-randomized designs provide less certainty of causality, and these findings should be tested in randomized trials in order to exclude potential unmeasured confounders. However, this study used prospective longitudinal data, controlled for baseline HIV risk behaviour and a range of potential confounders of both HIV risk and social protection access, and showed high external validity by testing the effects of government-led and administered cash/cash-in-kind programmes, NGO programming and care at the family and school levels. The study took place in a unique period in South Africa in which child-focused cash transfers were being expanded from an upper age limit of 14 to 18 and school feeding schemes were being expanded, thus allowing testing of national programmes that were large-scale but not yet universal. Therefore, these findings suggest effectiveness of social protection in real-world conditions in South Africa.

Another limitation is that all study sites were health districts with high HIV prevalence and thus in South Africa were high-poverty, African-majority areas [9]. Consequently, the findings cannot be generalized to wealthier areas or to other ethnic groups, nor to institutional settings that did not occur in our randomly selected communities, such as prisons. However, the sample included wide population variation, with urban/rural settings and five language groups in two provinces. A further limitation is that self-reported HIV risk behaviour should ideally be validated with biomarkers of HIV infection, although a number of systematic reviews have demonstrated strong associations between the self-reported sexual risk behaviours used in this study and HIV infection rates [i.e. 49]. An additional limitation is that it is likely that this study only measured some of the structural drivers, psychosocial mediators and social protection provisions that impact HIV risk. Whilst this study provides evidence of patterns of risk and protection for adolescents, future research should valuably examine other potential predictors along these pathways. Finally, this study had a follow-up of only one year. It would be valuable to test how social protection is associated with HIV risk behaviours over a young person's life cycle, from childhood to adulthood. Future research should also test the HIV-prevention effects of combining social protection and biomedical forms of HIV prevention.

Despite these limitations, this study provides valuable evidence for HIV prevention policy. Structural drivers put some adolescents in South Africa at high risk of infection, but risk pathways can be mitigated. Unconditional social protection is associated with reduced structural and psychosocial pathways to HIV risks. Perhaps most importantly, this study demonstrates that social protection may be most effective for those experiencing the most severe disadvantage. With the provision of real-world social protection, adolescents’ likelihoods of contracting HIV may not be inevitably defined by their socio-economic circumstances.
